# Zero-knowledge identity authentication for internet of vehicles: Improvement and application

**DOI:** 10.1371/journal.pone.0239043

**Published:** 2020-09-28

**Authors:** Mu Han, Zhikun Yin, Pengzhou Cheng, Xing Zhang, Shidian Ma

**Affiliations:** 1 School of Computer Science and Communication Engineering, Jiangsu University, Zhenjiang, China; 2 Automotive Engineering Research Institute Jiangsu University, Zhenjiang, China; University of Electronic Science and Technology of China, CHINA

## Abstract

The popularity of Internet of Vehicles (IoV) has made people's driving environment more comfortable and convenient. However, with the integration of external networks and the vehicle networks, the vulnerabilities of the Controller Area Network (CAN) are exposed, allowing attackers to remotely invade vehicle networks through external devices. Based on the remote attack model for vulnerabilities of the in-vehicle CAN, we designed an efficient and safe identity authentication scheme based on Feige-Fiat-Shamir (FFS) zero-knowledge identification scheme with extremely high soundness. We used the method of zero-one reversal and two-to-one verification to solve the problem that FFS cannot effectively resist guessing attacks. Then, we carried out a theoretical analysis of the scheme’s security and evaluated it on the software and hardware platform. Finally, regarding time overhead, under the same parameters, compared with the existing scheme, the scheme can complete the authentication within 6.1ms without having to go through multiple rounds of interaction, which reduces the additional authentication delay and enables all private keys to participate in one round of authentication, thereby eliminating the possibility that a private key may not be involved in the original protocol. Regarding security and soundness, as long as private keys are not cracked, the scheme can resist guessing attacks, which is more secure than the existing scheme.

## Introduction

The rise of IoV technology has not only changed the way people travel, but also made people's driving environment more and more comfortable [1]. However, the interconnection of networks between vehicles brings various information security, which makes the attack surface of the internal vehicle networks rise sharply, especially for remote attacks [2, 3]. In 2013, Toyota Prius cars were attacked by a hacker through the On-Board Diagnostic (OBD) interface and the braking systems were illegally manipulated, which caused traffic accidents [2]. In 2015, the 360 Crack team successfully cracked Build Your Dreams (BYD) and Tesla intelligent vehicles through remote and short-range attacks [4]. In the same year, Charlie Miller and Chris Valasek demonstrated the process of remotely attacking the Jeep Cherokee on-board system, including manipulation of speed, direction, brakes, and wipers [5]. In 2016, Tencent Keen Security Lab remotely reset the Bluetooth connection password of the Xiaomi Millet Nine Balancing Vehicle to achieve illegal manipulation. The following year, Tencent Keen Security Lab again found multiple high-risk vulnerabilities of security in Tesla's in-vehicle network [6]. In 2019, Tencent Cohen Lab can remotely gain the root privileges of the "Autopilot Electronic Control Unit (ECU)" module and control the steering system of the vehicle [7].

The above security issues are attributed to the lack of security protection mechanism in the traditional in-vehicle network [4]: 1) External devices have unrestricted access to in-vehicle data via wireless, Bluetooth, cellular network or OBD [8, 9]. 2) The information data of in-vehicle network are transmitted in the form of broadcast and plaintext, such as in the CAN bus. The broadcast data frame does not cover the source address and destination address [10]. Although [11–15] studied the security of in-vehicle networks to address these emerging issues, these in-vehicle security schemes focus on ensuring secure communication between ECUs with little consideration for the security issues introduced by external devices connected to the vehicle. The remote attacks on vehicles usually come from external networks or devices. If we only protect the vehicle network, such as data encryption, ECU authentication, data access control, which seems to be unable to play a decisive role, illegal devices can still inject malicious data frames into the vehicle network. Therefore, it is urgent to study the resistance to the invasion of external malicious nodes.

### Contributions

Identity authentication is very important as one of the important means to prevent external intrusion. However, we also had to consider the security of the authentication protocol, because an attacker can eavesdrop on valid authentication information in the authentication protocol to fake an identity. Therefore, we adopt the zero-knowledge identity authentication method to effectively solve the problem of proof information leakage in the prover's proof process. In this paper, based on the remote attack model for vehicles, we designed an efficient and safe identity authentication scheme based on FFS zero-knowledge identification scheme with extremely high soundness, which realizes the identification of the vehicles to the external devices and solves the security threat of unauthorized access and illegal intrusion. The main contributions of this paper are presented as follows:

1. Based on the analysis of existing attack events, we proposed a common remote attack model on vehicles and conduct a security threat assessment.
2. Based on the common remote attack model, we designed an efficient and safe identity authentication scheme based on FFS zero-knowledge identification scheme with extremely high soundness. The FFS scheme is based on the Quadratic Residue (QR) difficult problem. We improved the FFS scheme so that it can be applied to the IoV scenario that requires low latency and high security. We used the method of zero-one reversal and two-to-one verification to solve the problem that FFS cannot effectively resist guessing attacks. Therefore, it can meet extremely high soundness in one iteration of authentication.
3. We constructed a security architecture in a hardware environment and performed performance evaluation. According to the evaluation results, the scheme can complete the authentication without having to go through multiple rounds of interaction, which reduces the additional authentication delay and enables all private keys to participate in one round of authentication, thereby eliminating the possibility that a private key may not be involved in the original protocol. Regarding security and soundness, as long as private keys are not cracked, the scheme can resist guessing attacks. Therefore, the proposed scheme takes precedence over existing solutions in terms of time delay and security.

### Organization

The rest of this paper is organized as follows: In Section 2, we reviewed more related work. Section 3 presented some preliminary knowledge and analysis of FFS scheme. Section 4 presented the main remote attack model for the actual vehicle and the resulting security threat assessment. Section 5 introduces our scheme. In Section 6, we conducted a theoretical analysis of the security of the proposed architecture. In Section 7, we simulated and evaluated the performance of the proposed solution. Finally, Section 8 presented the summary of this study.

## Related work

Considering that the security of in-vehicle networks directly threatens the security of users' lives and property, the information security problems caused by external devices have to be solved. However, due to the low computing power of the ECU, solving the problems of vehicle network information security is still a huge challenge [1]. In order to resist forgery attacks, tampering attacks, replay attacks, and privacy leak attacks, researchers have studied many authentication schemes in the IoV or in-vehicle network [3, 12]. Research on authentication protocols based on privacy protection policies is the main method to ensure the integrity, reliability, and identity privacy of message transmission. It is also the basis for ensuring the security of information transmission in IoV. When any entity in the IoV receives relevant traffic messages, it must first pass authentication to ensure that the source of the message is reliable, the content is complete and authentic, has not been tampered with and replayed, and the identity of the user has not been leaked.

In order to solve the problem of certificate management in Public Key Infrastructure (PKI), Shamir [16] proposed identity-based cryptosystems in 1984. In this system, the identity information of each user can be used as the user's public key, such as e-mail name, phone number, ID number. The third-party trusted Public Key Generator (PKG) computes a private key based on the public key for each user and sends it to the user. Users can use the public and private keys in their hands for data encryption and digital signature operations. The cryptosystems provide data integrity mechanisms, digital envelopes, user identification, user authentication, and other technologies. On this basis, Shamir et al. [17, 18] proposed a zero-knowledge identity authentication scheme based on QR, but this scheme cannot effectively resist key guessing attacks. Kumari et al. [19] proposed an improved smart card based authentication scheme for session initiation protocol, which increases the probability of resisting key guessing attacks.

In the application scenario of IoV, Chim [20] proposed an identity-based authentication scheme, which is based on a bilinear pairing algorithm, can support batch authentication, and has low computing energy consumption. However, Horng et al. [21] believed that Chim's scheme could not resist forgery attacks and proposed a security scheme to overcome the problem of forgery attacks. However, since this scheme is based on bilinear pairing operations, its calculation time is three times that of ordinary dot multiplication operations [22]. Wang and Liu [23] proposed a certificate-based multi-level security authentication scheme that integrates all the inside and outside interfaces of the vehicles into the On Board Unit (OBU). However, in the system initialization of this scheme, a secure channel is used to transmit the symmetric key, and only the certificate is used for authentication during the two-way handshake authentication process. Therefore, the security of this solution is not high. Woo et al. [24] proposed to split the truncated MAC into the extended ID field and CRC field of the data frame, which can reduce the bus load, but it makes the data frame lack security and cannot verify the error during transmission. In addition, the scheme they proposed only had key negotiation for external devices and no identity verification, which greatly increased security risks. Li et al. [25] proposed a robust and energy-saving three-factor authentication protocol that can block the most common attacks and provide some ideal functions. The protocol reduces the power consumption and computational cost of nodes by using appropriate communication models and lightweight algorithms. Ying and NAYAK [26] proposed an anonymous lightweight authentication method based on smart card protocol, which uses low-cost encryption operations to verify the legitimacy of vehicles and data messages. From the above, these literatures use the identity-based key system in the IoV, combining the different characteristics of the IoV, to achieve the reliability and confidentiality of information transmission. Although many solutions can guarantee high security, most of them are not applicable in the scenario of fast connection authentication of the IoV. Therefore, it is of great research value to design a safe and effective authentication scheme for the connected vehicle application scenario.

## Background

### Quadratic residue problem

Definition 1 [27, 28]: Let *n* be a positive integer. If the congruence *x*^2^ ≡ *a* mod *n* have a solution and gcd (*a*, *n*) = 1, then *a* is called the quadratic residue of modulo *n*, where gcd means taking the greatest common divisor. Otherwise, *a* is called the quadratic non-residue of the module *n*.

The important conclusion about quadratic residue in number theory is given here directly: if *n* = *p* * *q*, where *p* and *q* are two prime numbers, a is the quadratic residue of modulo *n* if and only if formula (1) holds, where the symbol () in formula (1) is the Jacobi symbol. Based on this, the definitions of quadratic residue and pseudo-quadratic residue are given, as defined 2.

$$\left(\frac{a}{p}\right)=\left(\frac{a}{1}\right)=1$$ (1)

Definition 2 [27, 28]: *QR*(*n*) represents the set of quadratic residues of all modules *n* and $Q\tilde{R}\left(n\right)$ represents the set of pseudo quadratic residues of all modules *n*, and their definitions are as follows:

$$QR(n)=\left\{x\in Z_{n}:\left(\frac{x}{p}\right)=\left(\frac{x}{q}\right)=1\right\}$$ (2)

$$Q\tilde{R}(n)=\left\{x\in Z_{n}:\left(\frac{x}{p}\right)=\left(\frac{x}{q}\right)=-1\right\}$$ (3)

QR problem: from Definition 2 and Jacobi symbol correlation operation, it can be known that for any *x* ϵ *QR*(*n*), there is formula (4); if formula (5) holds, then *x* ϵ *QR*(*n*) or $x\in Q\tilde{R}\left(n\right)$. Only by knowing the values of *p* and *q* can you determine which set *x* belongs to. In other words, given a composite number *n* and an *x* (*x* ϵ Z_*n*_), *x* is determined to be the quadratic residue of the modulus *n*, where *n* is obtained by multiplying two large prime numbers *p* and *q*.

QR hypothesis: Let *F*_*QR*_ be a polynomial probability time efficient algorithm for solving QR problem. The *Adv*_*QR*_ = *Prob*[*F*_*QR*_(*n*,*x*) = *yes* (*x* ϵ *QR*(*n*))] is used to represent the probability that the algorithm *F*_*QR*_ solves the QR problem in polynomial time. If and only if there is no polynomial probability time algorithm which can solve the QR problem, the QR hypothesis is true (i.e., *Adv*_*QR*_ is negligible).

$$\left(\frac{x}{n}\right)=\left(\frac{x}{p}\right)\left(\frac{x}{q}\right)=1$$ (4)

$$\left(\frac{x}{n}\right)=1$$ (5)

### Analysis of feige-fiat-shamir identification scheme

**Fig 1:**
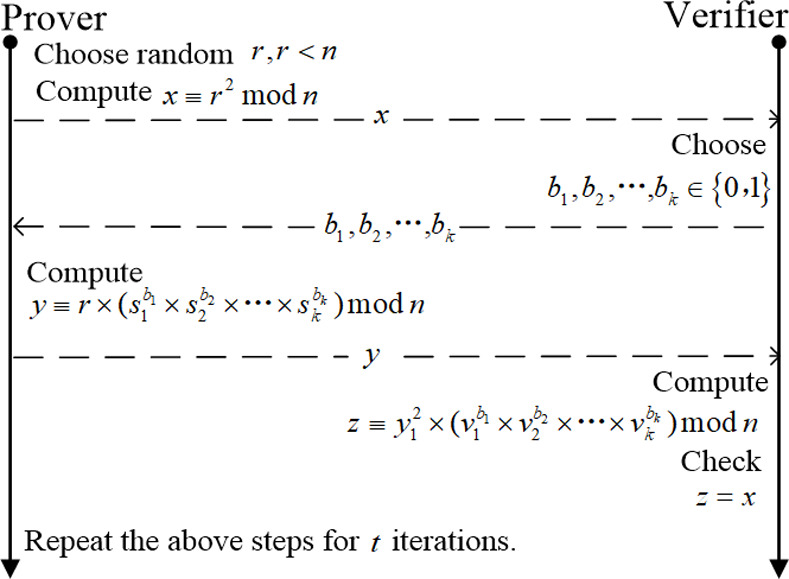
FFS identification scheme.

The Feige-Fiat-Shamir [17, 29, 30] identification scheme [30] is derived from the Fiat-Shamir [18] identification scheme, which is based on the intractability of computing square roots modulo n [29]. The parallel interactive mode’s process of the FFS identification scheme [31, 32] is shown in Fig 1.

**Fig 2:**
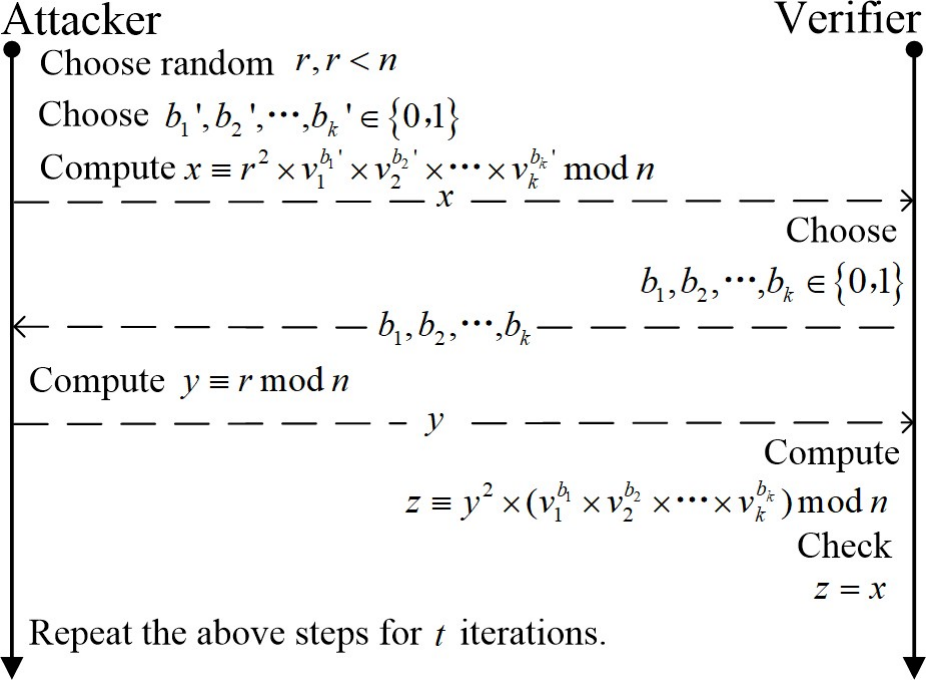
The attacker guesses $b_{i}{}^{\prime }$ in advance and hides the corresponding public key $v_{i}^{b_{i^{\prime }}}$ in the promise *x*. Then, ignore the received *b*_*i*_, and directly send the random number *r* as the response *y* to the verifier. Finally, if $b_{i}{}^{\prime }$ are equal to *b*_*i*_, the forgery is successful.

Attackers can use public keys to forge a promise to spoof. The forgery process is shown in Fig 2. FFS identification scheme is provably secure against chosen message attack in the following sense: provided that factoring n is difficult, the best attack has a probability 2^−kt^ of successful impersonation [29]. Choosing k and t such that k*t = 20 allows a 1 in a million chance of impersonation, which suffices in the case that an identification attempt requires a personal appearance by a would-be impersonator [29]. Specific parameter choices might be, for security 2^−20^: k = 5, t = 4. At present, the value of the number of bits of the parameter n requires more than 1024 bits in terms of calculation security [33]. Dhanya and Megha [33] Proposed an improved parallel interactive Feige-Fiat-Shamir identification scheme with almost zero soundness error and complete zero-knowledge. If the FFS authentication scheme is applied to an actual network communication environment, the security parameters need to be weighed. However, because of the high requirements for communication time in some application environments, these identification schemes seem not so suitable. To make identification schemes like FFS applicable to demanding application environments, further improvements are needed, which is also worth studying.

### Provable security

In 1984, Goldwasser and Mlicali [34] proposed provable security, which is an axiomatic research method and a new proof of security. The following security model will be used in section VI. Let *A* be the attacker and *C* be the challenger.

Initialization phase: *C* initializes the system and sends the public system parameters to *A*.

Phase 1: *A* makes a query for C (i.e., *A* sends ciphertext *c* to *C*, and *C* decrypts ciphertext c and sends the decrypted plaintext *m*_*b*_ to *A*).

Challenge: *A* outputs two equal-length plaintext messages *m*_0_ and *m*_1_, and then receives *m*_*b*_ ciphertext *c*_*b*_ from *C*, where *b* ϵ {0,1}.

Phase 2: *A* continues to repeat the process of Phase 1.

Guess: *A* outputs a random value *b’* ϵ {0,1}, and if *b*’ = b, *A* challenges successfully.

If the advantage of attacker A's successful attack in the probability polynomial time is negligible, the designed solution is safe.

## Attack model and security threat assessment

### Attack model

**Fig 3:**
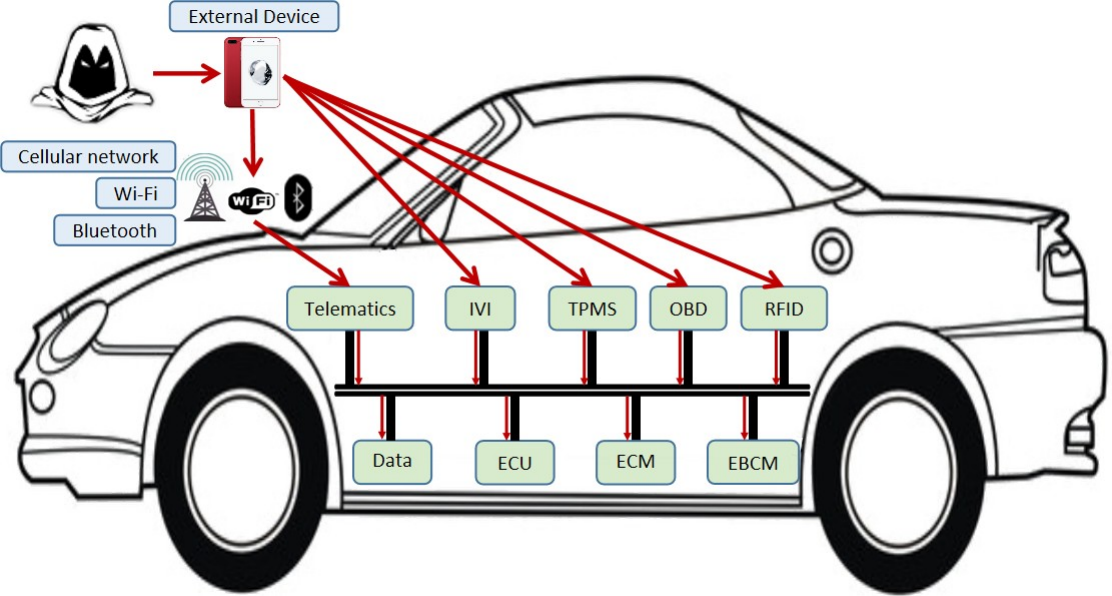
FFS identification scheme.

In reality, the attacker is more inclined to conduct remote attacks on vehicles. Vulnerabilities of in-vehicle networks include weak access control mechanisms, plaintext transmission and no identity authentication. If an attacker wants to control the vehicle through vulnerabilities of in-vehicle networks, he can pretend to be a legitimate external device, such as mobile phones and headphones, to deceive the vehicle communication unit, such as navigation systems and entertainment information systems. It is challenging to attack the in-vehicle CAN network through remote attacks. The attacker needs to first break through the vehicle communication unit, such as OBU and telematics. After successfully controlling the in-vehicle communication unit, they can steal and analyze the data in the CAN network, then fake the legitimate data frames, and inject them into the CAN network, which will cause great harm to the in-vehicle key ECUs [35]. Fig 3 presents the eight common attack surfaces and their attack processes. If the attacker is around the car, he can invade from the following attack surfaces including Wi-Fi, Cellular network, Car virtual key, Sensor and Bluetooth. If the attacker is sitting in the car, he can attack from the following attack surfaces including In-Vehicle Infotainment, USB, OBD-II. In Fig 3, although OBD and USB appear to be short-range attack surfaces, they can be combined with remote attack surfaces to attack [24]. We don't consider Denial of Service (DoS) attacks.

### Security threat assessment

When attackers implement the proposed attack model, the vehicle will face numerous threats as follows.

Eavesdropping attack: it mainly steals user privacy, communication units and data frames on the vehicle network, resulting in privacy information leakage.

Replay attack: The damaged external device repeatedly sends valid data to the communication units, which may affect the normal operation of the communication units. Data frames on the CAN network are repeatedly acquired and broadcast to the CAN network, which affects the normal operation of other ECUs.

Forgery attack: forge a legitimate external device or communication unit and broadcast malicious data frames to the CAN network to affect the normal operation of other ECUs.

Tampering attack: The data information between the external device and the communication units has been tampered with. tampering with eavesdropped data frames and broadcasting them to the CAN network, thereby deceiving the ECU.

If an illegal external device successfully deceives the in-vehicle communication unit, it will pose a huge threat to the vehicle and even threaten the safety of passengers' property and life. This is because external devices will attack the vehicle network through various vulnerabilities in the vehicle, such as broadcasting malicious data frames to the CAN network, and then ECUs will execute this fake instruction. Moreover, once the in-vehicle communication units are attacked, not only the vehicle networks will collapse, but also the vehicle ad hoc networks will be destroyed, which will also affect other vehicles or other legitimate external devices accordingly. According to the above attack model and evaluation results, security measures such as identity authentication of external devices and encryption of communication data should be adopted, so that combined with in-vehicle security protocols can effectively resist these threats.

## The proposed scheme

Zero-knowledge proof is that the prover can make the verifier believe that a certain conclusion is correct without providing the verifier with any useful information, which has better security. We adopted this method to hide the private key information of the prover. Furthermore, we chose the FFS zero-knowledge identity authentication based on QR, because the calculation amount of its one-round authentication is not particularly large, and it can be applied to ECUs whose computing power is not particularly high. However, the FFS scheme requires many rounds of certification, which limits its application to a certain extent, such as a high-velocity connected car environment. If the FFS scheme can be further optimized and improved, it would be better.

**Fig 4:**
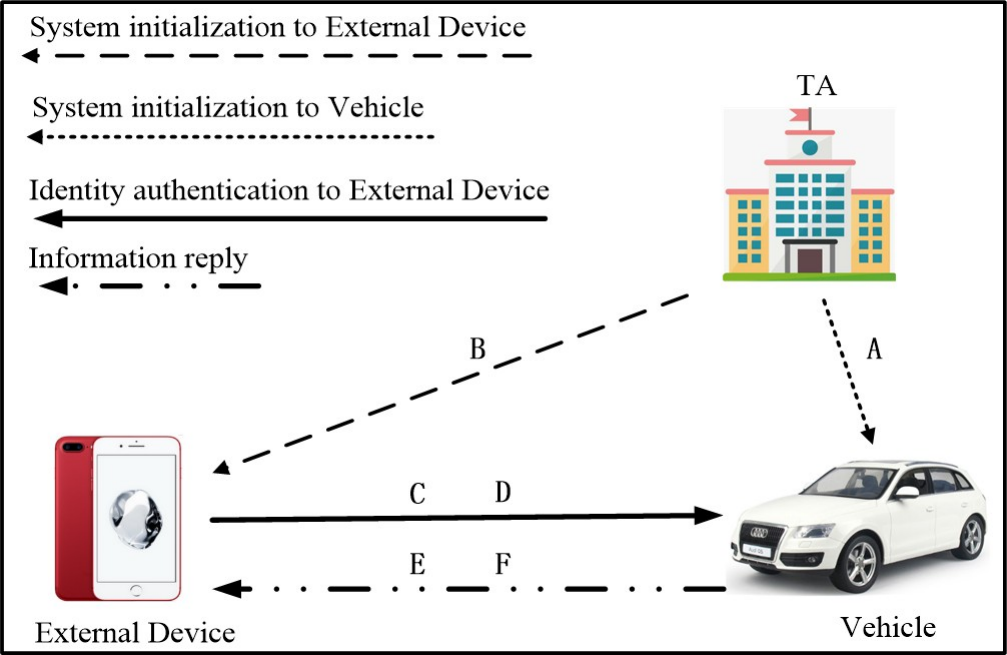
System model. A represents for the message sent by TA to Vehicle, including TA's public key *PK*_*TA*_ and parameters. B represents for the message sent by TA to ED, including signature *δ*_*EDv*_, ED's public key *v*_*i*_ and private key *s*_*i*_. C-F represents the messages passed in the process of identity authentication. The order of A-F is defined according to the order of events.

In order to better describe our protocols, the main notations in the scheme are summarized in the Table 1. As shown in Fig 4, the designed effective and safe identity authentication scheme mainly includes the following two parts:

System initialization: the Trusted Authority (TA) first initializes the system to calculate and disclose system parameters, and then distributes public-private key pairs and calculates identity authentication parameters for the mobile phone and vehicle-mounted terminals applying for registration.

Identity authentication: The proposed scheme is adopted between the mobile phone and the vehicle, which avoids sending certificates to trusted authorities, improves the efficiency of node identity authentication, and addresses privacy issues in the process.

**Table 1:** Notations used for protocol.

Notation	Description
*PK*_*TA*_	Public key of TA
*SK*_*TA*_	Private key of TA
*H*()	Hash function
*HMAC*()	Hash function value
*δ*_*EDv*_	Signature
*CTR*_*x*_	Message counter value of *x*
*E*()	Encryption function
*s*	The prover's private key
*v*	The prover's public key
*TS*	Time stamp
*ID*_*i*_	Identity of *i*
*l*_*x*_	Time to generate *x* in algorithm
*l*_*y*_	Time to generate *y* in algorithm
*l*_*h*_	Time to generate hash value

### System initialization

**Fig 5:**
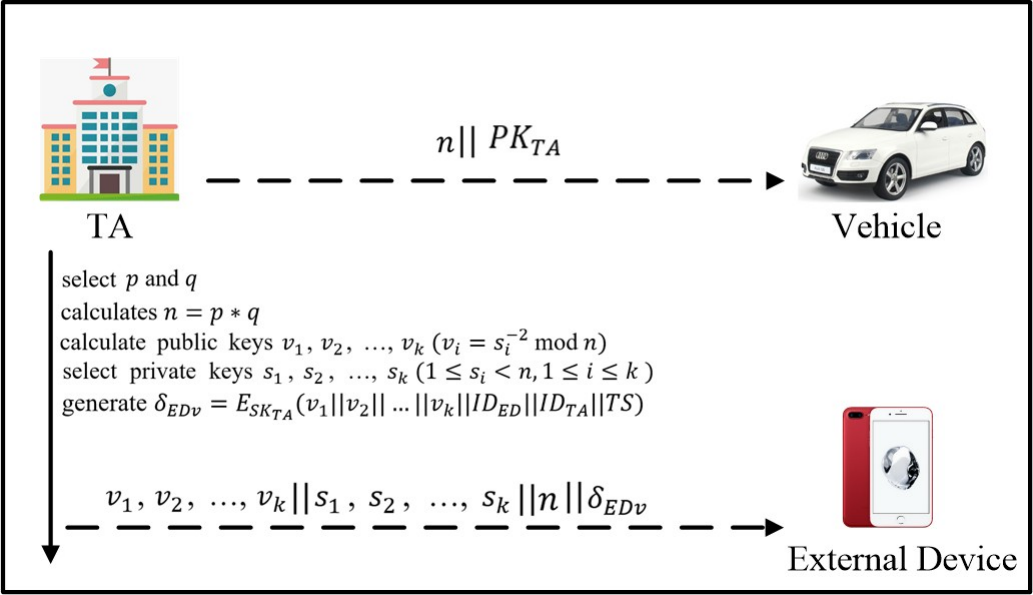
System initialization.

As shown in Fig 5, after the ED sends the registration request, TA selects two large prime numbers *p* and *q*, calculates *n* = *p* * *q*, and make the parameter *n* public. TA selects *k*(*k*≥2) random integers that are prime and different from each other as the private keys *s*_1_,*s*_2_, …,*s*_*k*_(1≤*s*_*i*_<,1≤*i*≤*k*), calculates the corresponding public keys $v_{1},v_{2},\;\ldots ,v_{k}(v_{i}=s_{i}^{-2}\;\mathrm{mod}\;n)$, and then signs the public keys to generate $\delta_{EDv}=E_{SK_{TA}}(v_{1}\left\|v_{2}\right\|\ldots \left\|v_{k}\right\|ID_{ED}\left\|ID_{TA}\right\|TS)$. Finally, these parameters are secretly sent to the registered external device. It is assumed that the registered external device and the vehicle have downloaded or saved the public key *PK*_*TA*_ of TA. It is assumed that the vehicle has already been registered. Here we only considered the detailed initialization of ED.

### Identity authentication

**Fig 6:**
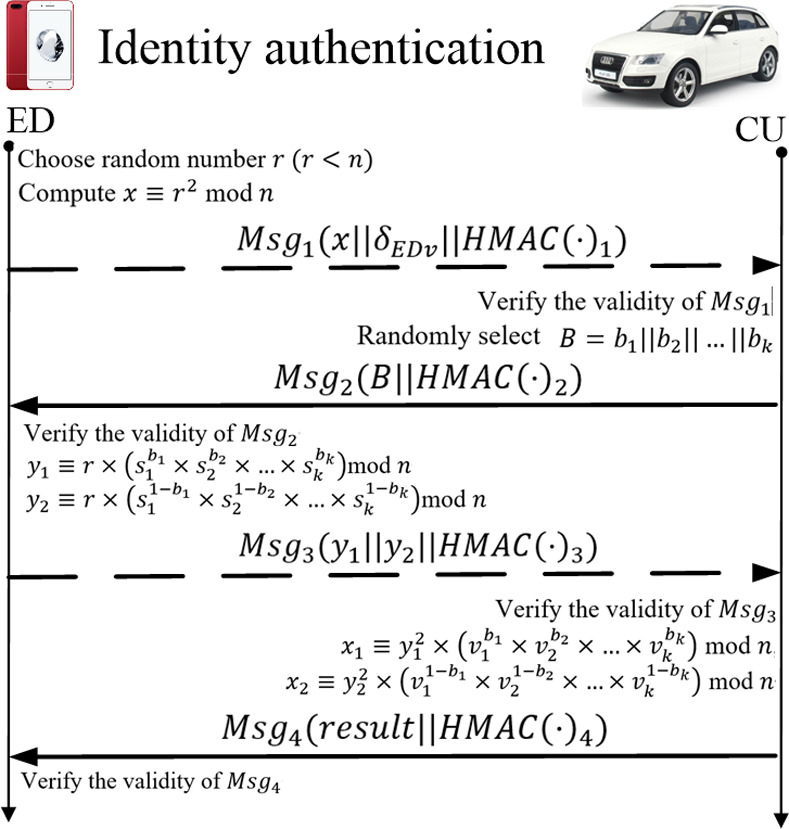
Identity authentication.

For a better description, the external device is referred to as ED, and the vehicle is referred to as CU (i.e., communication unit). After the system initialization, we proposed two schemes for CU to ED authentication. As shown in Algorithm 1 and Fig 6, the basic scheme is that CU authenticates the identity of ED for the first time. Concrete steps are as follows.

Step 1: ED chooses random number *r*, where *r*<*n*, and compute *x* by (6) and *HMCA*(·)_1_ by (7), Then, ED sends *Msg*_1_(*x*∥*δ*_*EDv*_∥*HMCA*(·)_1_) to CU. ED increases *CTR*_*ED*_ by 1.

$$x\equiv r^{2}\;\text{mod}\;n$$ (6)

$$HMCA{\left(\cdot \right)}_{1}=H(CTR_{ED}\left\|x\right\|ID_{ED})$$ (7)

Step 2: After receiving message *Msg*_1_, CU uses the hash function *H*() to generate $HMCA{\left(\cdot \right)}_{1}^{*}$ for *CTR*_*ED*_, *x*, and *ID*_*ED*_ by (8), and compares it with *HMCA*(·)_1_ in *Msg*_1_ to ensure the validity of *Msg*_1_. After successful verification, CU decrypts *δ*_*EDv*_ in *Msg*_1_ by *PK*_*TA*_ and check *ID*_*ED*_∥*ID*_*TA*_∥*TS*. If *ID*_*ED*_∥*ID*_*TA*_∥*TS* are valid, CU saves *ID*_*ED*_ and *v*_*1*_∥*v*_*2*_∥…*v*_*k*_ to the secure storage. Further, CU randomly selects k-bit binary bit strings *b*_*1*_∥*b*_*2*_∥…*b*_*k*_ and compute *HMCA*(·)_2_ by (9). Finally, CU sends *Msg*_2_(*B*∥*HMCA*(·)_2_∥) to ED, where *B* is *b*_*1*_∥*b*_*2*_∥…*b*_*k*_. CU increases *CTR*_*ED*_ and *CTR*_*CU*_ by 1.

$$HMAC{\left(\cdot \right)}_{1}^{*}=H(CTR_{ED}||x||ID_{ED})$$ (8)

$$HMAC{\left(\cdot \right)}_{2}=H(CTR_{CU}||B||ID_{CU})$$ (9)

Step 3: After receiving message *Msg*_2_, ED uses the hash function *H*() to generate $HMCA{\left(\cdot \right)}_{2}^{*}$ for *CTR*_*CU*_, *B*, and *ID*_*CU*_ by (10), and compares it with *HMCA*(·)_2_ in *Msg*_2_ to ensure the validity of *Msg*_2_. After successful verification, ED computes *y*_1_ by (11), *y*_2_ by (12) and *HMCA*(·)_3_ by (13). Finally, ED sends *Msg*_3_(*y*_1_∥*y*_2_∥*HMCA*(·)_3_) to CU. ED increases *CTR*_*ED*_ and *CTR*_*CU*_ by 1.

$$HMAC{\left(\cdot \right)}_{2}^{*}=H(CTR_{CU}||B||ID_{CU})$$ (10)

$$y_{1}\equiv r\times (s_{1}^{b_{1}}\times s_{2}^{b_{2}}\times \ldots \times s_{k}^{b_{k}})\mathrm{mod}\;n$$ (11)

$$y_{2}\equiv r\times (s_{1}^{1-b_{1}}\times s_{2}^{1-b_{2}}\times \ldots \times s_{k}^{1-b_{k}})\text{mod}\;n$$ (12)

$$HMAC{\left(\cdot \right)}_{3}=H(CTR_{ED}\left\|y_{1}\right\|\left\|y_{2}\right\|ID_{ED})$$ (13)

Step 4: After receiving message *Msg*_3_, CU uses the hash function *H*() to generate $HMCA{\left(\cdot \right)}_{3}^{*}$ for *CTR*_*ED*_, *y*_1_, *y*_2_ and *ID*_*ED*_ by (14), and compares it with *HMCA*(·)_3_ in *Msg*_3_ to ensure the validity of *Msg*_3_. After successful verification, CU compute *x*_1_ and *x*_2_ by (15) and (16). If *x* = *x*_1_ =*x*_2_, ED is successfully authenticated by CU and CU sends *Msg*_4_(*result*∥*HMCA*(·)_4_) to ED, where *result* is 1 and *HMCA*(·)_4_ is computed by (17). Otherwise, ED is not authenticated by CU and CU sends *Msg*_4_(*result*∥*HMCA*(·)_4_) to ED, where *result* is 0 and *HMCA*(·)_4_ is computed by (12). CU increases *CTR*_*ED*_ and *CTR*_*CU*_ by 1.

$$HMAC{\left(\cdot \right)}_{3}^{*}=H(CTR_{ED}||y_{1}||y_{2}||ID_{ED})$$ (14)

$$x_{1}\equiv y_{1}^{2}\times (v_{1}^{b_{1}}\times v_{1}^{b_{2}}\times \ldots \times v_{k}^{b_{k})})\text{mod}\;n$$ (15)

$$x_{2}\equiv y_{2}^{2}\times (v_{1}^{1-b_{1}}\times v_{1}^{1-b_{2}}\times \ldots \times v_{k}^{1-b_{k}})\text{mod}\;n$$ (16)

$$HMAC{\left(\cdot \right)}_{4}=H(CTR_{CU}\left\|result\right\|ID_{CU})$$ (17)

Step 5: After receiving message *Msg*_4_, ED uses the hash function *H*() to generate $HMCA{\left(\cdot \right)}_{4}^{*}$ for *CTR*_*ED*_, *y*_1_,*y*_2_ and *ID*_*ED*_ by (18), and compares it with *HMCA*(·)_4_ in *Msg*_4_ to ensure the validity of *Msg*_4_. After successful verification, if *result* is 1, ED is successfully authenticated by CU. Otherwise, ED is not authenticated by CU. ED increases *CTR*_*CU*_ by 1.

$$HMAC{\left(\cdot \right)}_{4}^{*}=H(CTR_{CU}||result||ID_{CU})$$ (18)

## Algorithm 1 Authentication Protocol

1: ED: Choose random number *r*(*r*<*n*)

  Compute *x* = *r*^2^ mod *n*

 ED➜CU:*Msg*_1_(*x*∥*δ*_*EDv*_∥*HMCA*(·)_1_)

  Where *HMCA*(·)_1_ =*H*(*CTR*_*ED*_∥*x*∥*ID*_*ED*_)

 *CTR*_*ED*_++

2: CU: Verify the validity of *Msg*_1_

 If *HMCA*(·)_1_ are valid then

  Decrypt *δ*_*EDv*_ in *Msg*_1_ by *PK*_*TA*_ and check *ID*_*ED*_∥*ID*_*TA*_∥*TS*

  If *ID*_*ED*_∥*ID*_*TA*_∥*TS* are valid, then Obtain *v*_*1*_∥*v*_*2*_∥…*v*_*k*_

   Randomly select k-bit binary bit strings *b*_*1*_∥*b*_*2*_∥…*b*_*k*_

   CU➜ED: *Msg*_2_(*B*∥*HMCA*(·)_2_∥)

    Where *B* = *b*_*1*_∥*b*_*2*_∥…*b*_*k*_,

     *HMCA*(·)_2_ =*H*(*CTR*_*CU*_∥*B*∥*ID*_*CU*_)

   *CTR*_*CU*_++, *CTR*_*ED*_++

  else Refuse the request information

 else Refuse the request information

 endif

3: ED: Verify the validity of *Msg*_2_

 if *HMCA*(·)_2_ are valid then Compute

$$y_{1}\equiv r\times (s_{1}^{b_{1}}\times s_{2}^{b_{2}}\times \ldots \times s_{k}^{b_{k}})\text{mod}\;n$$

$$y_{2}\equiv r\times (s_{1}^{1-b_{1}}\times s_{2}^{1-b_{2}}\times \ldots \times s_{k}^{1-b_{k}})\text{mod}\;n$$

  ED➜CU:*Msg*_3_(*y*_1_∥*y*_2_∥*HMCA*(·)_3_∥) 

   Where *HMCA*(·)_2_ =*H*(*CTR*_*ED*_∥*y*_*1*_∥*y*_*2*_∥*ID*_*ED*_)

  *CTR*_*CU*_ ++, *CTR*_*ED*_++

 else Refuse the information

 endif

4: CU: Verify the validity of *Msg*_3_

 if *HMCA*(·)_3_ are valid then Compute

$$x_{1}\equiv y_{1}^{2}\times (v_{1}^{b_{1}}\times v_{2}^{b_{2}}\times \ldots \times v_{k}^{b_{k}})\text{mod}\;n,$$

$$x_{2}\equiv y_{2}^{2}\times (v_{1}^{1-b_{1}}\times v_{2}^{1-b_{2}}\times \ldots \times v_{k}^{1-b_{k}})\text{mod}\;n$$

  if *x* = *x*_1_ = *x*_2_ then

   CU➜ED: *Msg*_4_(*result*∥*HMCA*(·)_4_∥)

    Where *result* = 1 and *HMCA*(·)_4_ =*H*(*CTR*_*CU*_∥*result*∥*ID*_*CU*_)

  else CU➜ED: *Msg*_4_(*result*∥*HMCA*(·)_4_∥)

    Where *result* = 0 and *HMCA*(·)_4_ =*H*(*CTR*_*CU*_∥*result*∥*ID*_*CU*_)

   *CTR*_*CU*_ ++, *CTR*_*ED*_++

 else Refuse the information

 endif

5: ED: Verify the validity of *Msg*_4_

  if *HMCA*(·)_4_ are valid and *result* = 1

  then ED is successfully authenticated by CU

 else ED is not authenticated by CU.

 *CTR*_*CU*_ ++

endif

The enhanced scheme is based on the basic scheme. When the CU authenticates the ED for the first time, the information such as the ID of the ED and the public key *v*_*1*_∥*v*_*2*_∥…*v*_*k*_ has been stored by CU. In future identity authentication, it is not necessary to send the signature *δ*_*EDv*_ in Algorithm 1, and Other processes remain unchanged. It is worth noting that the calculation overhead of the enhanced scheme is less than the calculation overhead of the basic scheme, which saves authentication time.

## Security analysis of the proposed protocol

In order to verify the security of this scheme, in this section, we present the following theoretical proof.

Theorem 1. If the QR problem is difficult and the assumption is true, the proposed protocol can guarantee the security of the private key *s* and prevent the leakage of information (i.e., the probability that the attacker *A* calculates a valid private key from the public key is exceedingly negligible).

Proof 1. It is assumed that *A* can construct the algorithm *F*_*QR*_ to solve the QR difficulty problem. The advantage of *A*’s successful attack on *x* is defined as $Adv_{A}^{x}$.

*F*_*QR*_ publishes the public parameter {*n*, *v*, *H*} and saves the public-private key pairs: $s_{F}\in Z_{n}^{*}$, $v_{F}=s_{F}^{-2}\;\text{mod}\;n$. *A* can query *F*_*QR*_ for *q*_*QR*_ times at most.

Query: *A* makes queries on random number and key. Then, *F*_*QR*_ returns *x* = *r*^2^ mod *n* and *v* = *s*^*-*2^ mod *n* to *A*.

Challenge: *A* uses *F*_*QR*_ to get *r* = *F*_*QR*_(*x*, *n*) and *s* = *F*_*QR*_(*v*, *n*), respectively. That is given *x* and *n*, and *r* is obtained by computing.

The advantages of four successful challenges in this process are $Adv_{A}^{r}=q_{QR}*Adv_{QR}^{r}$ and $Adv_{A}^{s}=q_{QR}*Adv_{QR}^{s}$, respectively. According to the difficult problem mentioned above, the advantage $Adv_{A}^{x}$ of the algorithm *F*_*QR*_ successfully solving the QR difficulty problem in the polynomial time is negligible. Therefore, the attacker *A* cannot obtain random number *r* and key *s* in *x* = *r*^2^ *modn* and *v* = *s*^*-*2^ *modn*.

Theorem 2. Our protocol can guarantee the confidentiality and integrity of the message *m*, which can prevent information tampering attack, information disclosure and forgery attack.

Proof 2. Our protocol uses hash function *H*(*m*) to realize the confidentiality and integrity of the message *m*. In order to prevent the attacker from forging legal data frames, we add the counter *CTR*_*x*_ and identity *ID*_*x*_ to the *H*() function.

Theorem 3. Our scheme meets completeness, which meaning that honest verifier always accepts proof from honest prover.

Proof 3. Since our scheme is based on FFS, it also has completeness property.

Theorem 4. Our scheme meets soundness, which means that honest verifier never accepts proof from cheating prover since it is computationally more secure than the original FFS. In other words, as long as the number of bits of large composite number n meets the required security requirements, our scheme can almost resist guessing attacks.

Proof 4. If the attacker wants to forge a legitimate identity with public key $v_{i}^{b_{i^{\prime }}}$ to cheat the verifier, he needs to compute responses *y*_1_ and *y*_2_. Suppose the attacker has computed *x* in (14), and $b_{i}{}^{\prime }$ are equal to *b*_*i*_. Naturally *y*_1_ is equal to *r* in (20). Next, compute *y*_2_, which means that compute *X* in (21). Then, *x* and *y*_2_ are substituted into Eq (16) to compute *X* in (22). It can be seen that because of *b*_*i*_ ϵ {0,1}, the result of *X* is multiplied by these private keys *s*_*i*_ or their inverses $s_{i}^{-1}$. The QR difficulty problem has been mentioned in the previous section. It is obvious here that the attacker cannot compute *y*_2_, which means that the probability of successful impersonation of the best attack depends on the difficulty of factoring *n* rather than 2^-kt^.

$$x\equiv r^{2}\times (v_{1}^{b_{1^{\prime }}}\times v_{2}^{b_{2^{\prime }}}\times \ldots \times v_{k}^{b_{k^{\prime }}})\text{mod}\;n$$ (19)

$$y_{1}\equiv r\mathrm{mod}\;n$$ (20)

$$y_{2}\equiv r\times X\mathrm{mod}\;n$$ (21)

$$X\equiv \sqrt{\frac{v_{1}^{b_{1^{\prime }}}\times v_{2}^{b_{2^{\prime }}}\times \ldots \times v_{k}^{b_{k^{\prime }}}}{v_{1}^{1-b_{1}}\times v_{2}^{1-b_{2}}\times \ldots \times v_{k}^{1-b_{k}}}}\;\mathrm{mod}\;n$$

$$\equiv \sqrt{\frac{s_{1}^{-b_{1}}\times s_{2}^{-b_{2}}\times \ldots \times s_{k}^{-b_{k}}}{v_{1}^{1-b_{1}}\times v_{2}^{1-b_{2}}\times \ldots \times v_{k}^{1-b_{k}}}}\;\mathrm{mod}\;n$$

$$\equiv \prod_{i=1}^{k}s_{i}^{1-2b_{i}}\;\mathrm{mod}\;n$$ (22)

Theorem 5. Our scheme meets zero-knowledge, which meaning that cheating verifier is never able to learn the prover’s secret.

Proof 5. The method of proof is to construct a simulator *Sim* with the same computing resources as Verifier (V), which is indistinguishable from the real authentication process in polynomial time. *Sim* is used to generate legal interactive content.

Query 1: *Sim* makes queries, randomly chooses *x*, and sends *x* to V. Then, Verifier returns *B* = *b*_1_∥*b*_2_∥…∥*b*_*k*_ to *Sim*.

Challenge 1: *Sim* randomly chooses *r*, computes $x\equiv \frac{r^{2}}{v_{1}^{b_{1}}\times v_{2}^{b_{2}}\times \ldots \times v_{k}^{b_{k}}}\text{mod}\;n$.

Query 2: *Sim* interacts with V again and sends *x* of Challenge 1 to V. Then, V returns *B* = *b*_1_∥*b*_2_∥…∥*b*_*k*_ to *Sim*.

Challenge 2: *Sim* sends *r* of Challenge 1 to V.

Obviously, the output $y_{1}{}^{\prime }$ and $y_{2}{}^{\prime }$ of *Sim* and the output *y*_1_ and *y*_2_ of V are the same distribution, which are indistinguishable in polynomial time. When *B* = 0 and *k* > 1, V will get *z* = *s*_1_×*s*_2_×*s*_*k*_ mod *n*. According to the difficult problem mentioned above and Proof 1, the advantage $Adv_{Verifier}^{s}$ of the algorithm *F*_*DL*_ successfully solving the QR difficulty problem in the polynomial time is negligible. Obviously, apart from convincing *Sim*, V cannot get any valuable information. Therefore, the scheme has zero-knowledge property under parallel composition.

## Simulation evaluation

**Fig 7:**
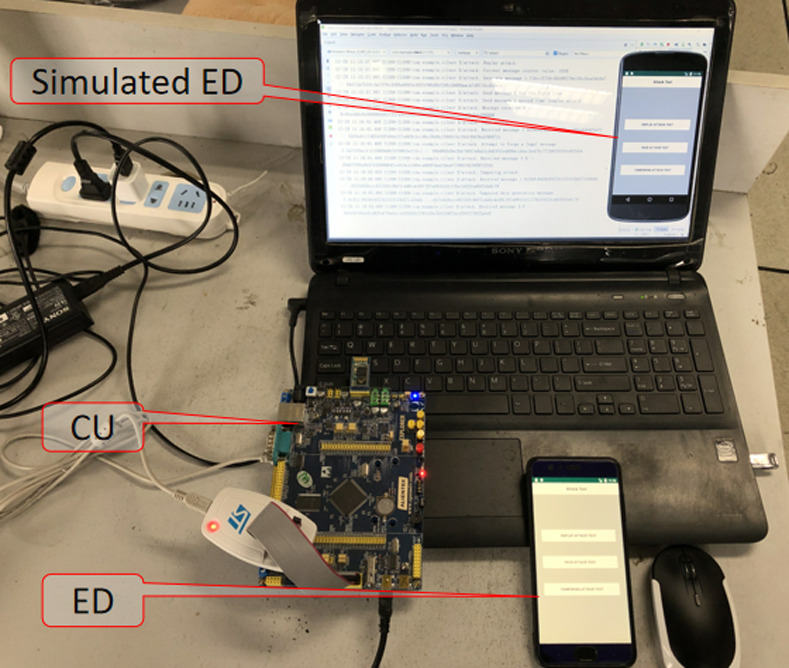
Hardware experimental environment.

In this section, to evaluate the performance and security of the protocols, we constructed a hardware experimental environment, which can be found in Fig 7. This experiment used STMicroelectronics' automotive microcontrollers, which is a high-performance 32-bit ARM Cortex®-M7 MCU developed by ST. The maximum operating frequency is 400MHz. We used a CU with a Bluetooth serial port and connected the CU to the ED via a Bluetooth module. The specifications of the tools employed in the hardware and software in the experiment are shown in Table 2. Firstly, we ported Contiki to Keil's MDK5 integration environment to compile the code used in the experiment and import the code into processors using ST-LINK V2. Secondly, we used Android to develop a simulated mobile phone on the computer as an ED and developed a corresponding Application (APP) for the proposed scheme to facilitate the implementation of the authentication. The simulated mobile phone system can be implanted on Android phones. Finally, we create Bmob cloud users based on JavaEE(SpringMVC) as a third-party trusted TA. In addition, we use JavaEE(SpringMVC) to develop a server-side to observe identity authentication process of the simulated CU and ED.

**Table 2:** Notations used for protocol.

Tools	Remarks
CU	STM32, 400MHz
Complier	Keil uVision5 (MDK5)
Software	JavaEE(SpringMVC)
PC	Used to install these software packages
ED	Android mobile phone
Bluetooth	Bluetooth 2.0
TA	Bmob cloud

**Fig 8:**
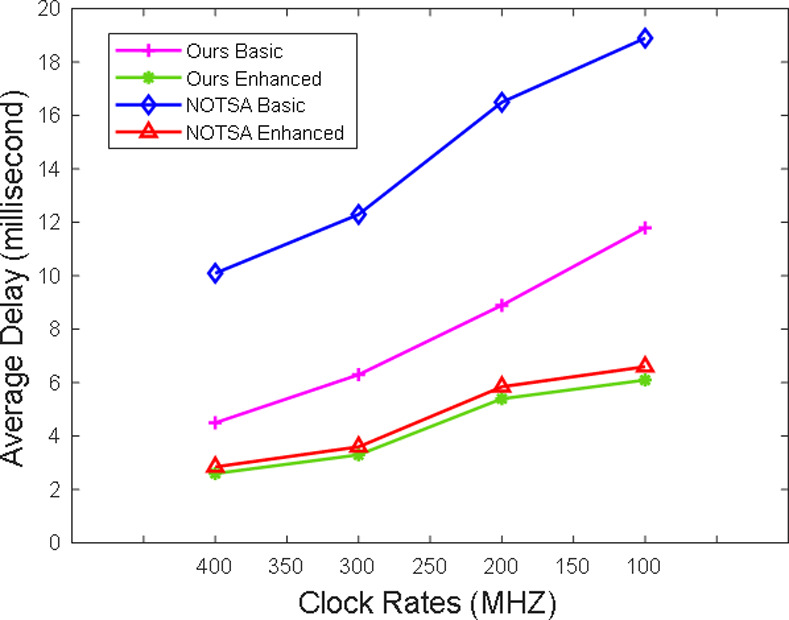
Comparison among ours and NOTSA at different clock rates.

In order to make our protocol more advantageous, we compared the average time delays of authentication at different clock rates (400, 300, 200, 168, 150, and 120 MHz) with the average time delays of NOTSA's authentication [23] and POSTER's authentication [36]. Compared with NOTSA, the proposed scheme has a smaller calculation amount, so the average time delays of ours are shorter, as shown in Fig 8. At different clock rates, the average time delays of our enhanced scheme are under 6.1 ms at different clock rates. In addition, average time delays of POSTER's the zero-knowledge proofs of are between 75–217 ms, which is much more than ours.

**Fig 9:**
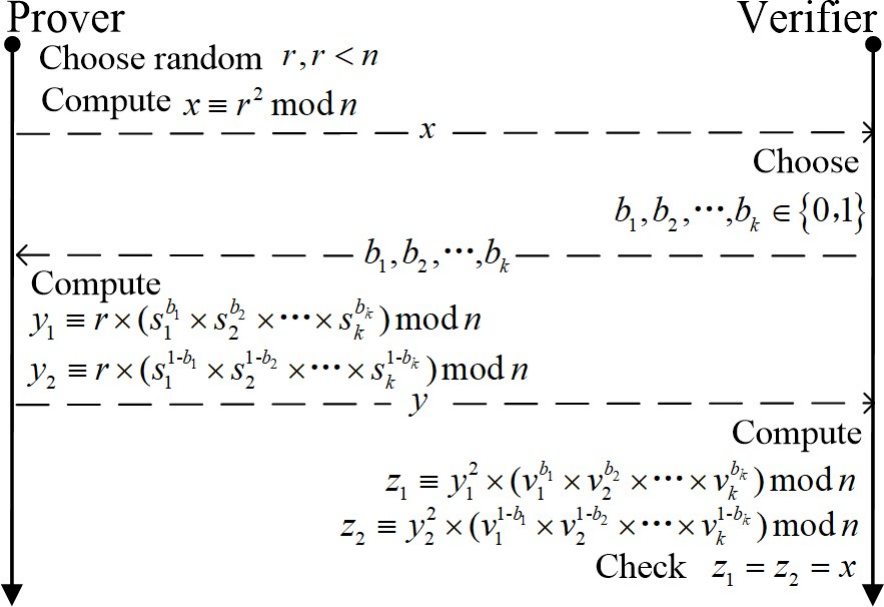
The proposed scheme with removing the hash function, counter value, etc.

**Fig 10:**
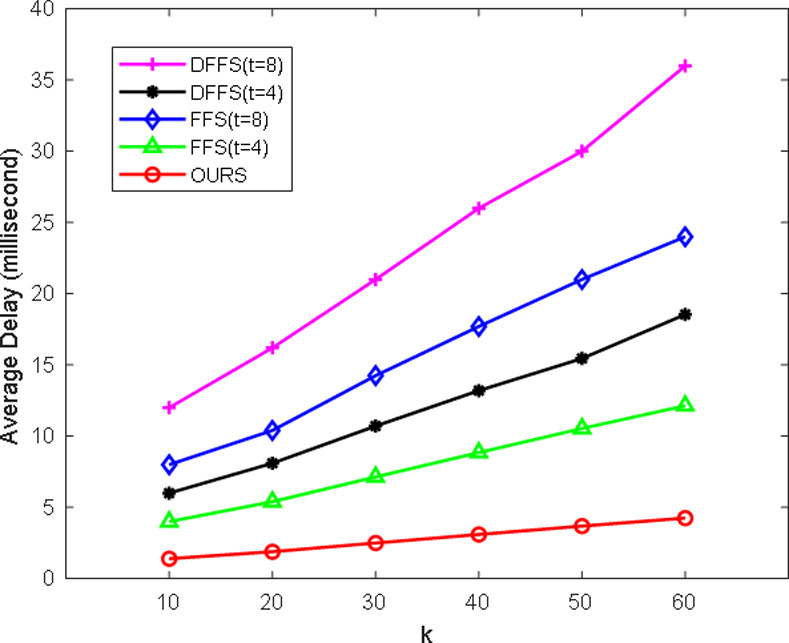
Comparison among DFFS, FFS, and ours at different values of k and t.

Simplify our scheme into the form of FFS scheme (e.g., remove the hash function, counter value, etc.), as shown in Fig 9. Then, the simplified scheme is compared with FFS and DFFS [33]. In order to make experimental data conveniently, we did simulation experiments on PC and compare the average delay of each scheme at different values of k and t, as shown in Fig 10. In the FFS scheme, the probability of soundness is 2-kt, which is obviously based on the size values of the k and t parameters. When the value of t is larger, the more times the prover interacts with the verifier, the longer the authentication delay. When the value of k is larger, the number of public and private keys stored is larger, and the harder it is to save and process. From the above section, it is worth mentioning that our scheme can achieve extremely high soundness with only one iteration of authentication, which is based on the QR difficult problem. In the DFFS scheme, it is an improved 3-pass parallel interactive scheme with ‘almost’ zero soundness error, which is based on FFS digital signature. The probability of soundness error (or cheating probability) is 2^-(kt+h/2)^. The DFFS scheme requires encryption and decryption of hash and the promised x value for each iteration of authentication. When the value of k is too large, especially when it is greater than 100, its authentication time delays will increase exponentially. Although the cheating probability is reduced, the computational overhead is increased, and the number of authentication iterations is still t. In our scheme, the idea of "two-to-one" (e.g., *y*_1_ and *y*_2_ correspond to *x*) and "reversal" (e.g., *b* and 1−*b*) can make it possible to have extremely high security in one iteration of authentication. In order to make the comparison schemes more impressive, a computational costs comparison table is given, as shown in Table 3. Therefore, our proposed scheme has higher security and efficiency, and it can meet extremely high soundness in one iteration of authentication.

**Table 3:** Computational costs comparison.

Scheme	FFS	DFFS	OURS
n (bit)	1024	1024	1024
The number of keys *s*	k	k	k
Number of authentication rounds	t	t	1
Is there a hash function?	No	SHA1	No
Communication overhead	*t*(2048 + *k*)	*t*(2048 + *k*)	3072 + *k*
Computational overhead	*t*(*l*_*x*_ + 2*l*_*y*_)	*t*(*l*_*x*_ + 4*l*_*y*_ + 2*l*_*h*_)	*l*_*x*_ + 4*l*_*y*_

## Conclusions

In this paper, we present the main attack models and security threat assessments for vehicles. Then, on this basis, we designed an efficient and safe identity authentication scheme based on Feige-Fiat-Shamir identification scheme with extremely high soundness. The proposed scheme has extremely high soundness based on the quadratic residue (QR) difficult problem. Subsequently, we analyzed the security of the proposed solution through a safety certificate. In simulation and evaluation, we built a hardware lab environment to evaluate performance. At the same time, software simulation was performed using JavaEE (SpringMVC) and Android. The experimental results prove that the proposed scheme is feasible and highly effective. In the future, the number of nodes in the Internet of Vehicles will increase rapidly. If each identity authentication is interactive, there may be some delays, which does not meet the high speed requirements of the Internet of Vehicles. Therefore, we will further study non-interactive zero-knowledge proofs for more secure and efficient authentication.

## Supporting information

S1 File (RAR)

S2 File (RAR)

S3 File (JAVA)

S4 File (JAVA)
